# High SUVs Have More Robust Repeatability in Patients with Metastatic Prostate Cancer: Results from a Prospective Test-Retest Cohort Imaged with ^18^F-DCFPyL

**DOI:** 10.1155/2022/7056983

**Published:** 2022-02-23

**Authors:** Rudolf A. Werner, Bilêl Habacha, Susanne Lütje, Lena Bundschuh, Takahiro Higuchi, Philipp Hartrampf, Sebastian E. Serfling, Thorsten Derlin, Constantin Lapa, Andreas K. Buck, Markus Essler, Kenneth J. Pienta, Mario A. Eisenberger, Mark C. Markowski, Laura Shinehouse, Rehab AbdAllah, Ali Salavati, Martin A. Lodge, Martin G. Pomper, Michael A. Gorin, Ralph A. Bundschuh, Steven P. Rowe

**Affiliations:** ^1^Department of Nuclear Medicine, University Hospital Würzburg, Würzburg, Germany; ^2^Comprehensive Heart Failure Center University Hospital Würzburg, Würzburg, Germany; ^3^Department of Nuclear Medicine, University Hospital Bonn, Bonn, Germany; ^4^Okayama University Graduate School of Medicine, Dentistry and Pharmaceutical Sciences, Okayama, Japan; ^5^Medical School Hannover, Department of Nuclear Medicine, Hannover, Germany; ^6^Nuclear Medicine, Medical Faculty, University of Augsburg, Germany; ^7^The James Buchanan Brady Urological Institute and Department of Urology, Johns Hopkins University School of Medicine, Baltimore, MD, USA; ^8^Sidney Kimmel Comprehensive Cancer Center, Johns Hopkins University School of Medicine, Baltimore, MD, USA; ^9^The Russell H. Morgan Department of Radiology and Radiological Science, Johns Hopkins University School of Medicine, Baltimore, MD, USA; ^10^Urology Associates and UPMC Western Maryland, Cumberland, MD, USA; ^11^Department of Urology, University of Pittsburgh School of Medicine, Pittsburgh, PA, USA

## Abstract

**Objectives:**

In patients with prostate cancer (PC) receiving prostate-specific membrane antigen- (PSMA-) targeted radioligand therapy (RLT), higher baseline standardized uptake values (SUVs) are linked to improved outcome. Thus, readers deciding on RLT must have certainty on the repeatability of PSMA uptake metrics. As such, we aimed to evaluate the test-retest repeatability of lesion uptake in a large cohort of patients imaged with ^18^F-DCFPyL.

**Methods:**

In this prospective, IRB-approved trial (NCT03793543), 21 patients with history of histologically proven PC underwent two ^18^F-DCFPyL PET/CTs within 7 days (mean 3.7, range 1 to 7 days). Lesions in the bone, lymph nodes (LN), and other organs were manually segmented on both scans, and uptake parameters were assessed (maximum (SUV_max_) and mean (SUV_mean_) SUVs), PSMA-tumor volume (PSMA-TV), and total lesion PSMA (TL-PSMA, defined as PSMA − TV × SUV_mean_)). Repeatability was determined using Pearson's correlations, within-subject coefficient of variation (wCOV), and Bland-Altman analysis.

**Results:**

In total, 230 pairs of lesions (177 bone, 38 LN, and 15 other) were delineated, demonstrating a wide range of SUV_max_ (1.5–80.5) and SUV_mean_ (1.4–24.8). Including all sites of suspected disease, SUVs had a strong interscan correlation (*R*^2^ ≥ 0.99), with high repeatability for SUV_mean_ and SUV_max_ (wCOV, 7.3% and 12.1%, respectively). High SUVs showed significantly improved wCOV relative to lower SUVs (*P* < 0.0001), indicating that high SUVs are more repeatable, relative to the magnitude of the underlying SUV. Repeatability for PSMA-TV and TL-PSMA, however, was low (wCOV ≥ 23.5%). Across all metrics for LN and bone lesions, interscan correlation was again strong (*R*^2^ ≥ 0.98). Moreover, LN-based SUV_mean_ also achieved the best wCOV (3.8%), which was significantly reduced when compared to osseous lesions (7.8%, *P* < 0.0001). This was also noted for SUV_max_ (wCOV, LN 8.8% vs. bone 12.0%, *P* < 0.03). On a compartment-based level, wCOVs for volumetric features were ≥22.8%, demonstrating no significant differences between LN and bone lesions (PSMA-TV, *P* =0.63; TL-PSMA, *P* =0.9). Findings on an entire tumor burden level were also corroborated in a hottest lesion analysis investigating the SUV_max_ of the most intense lesion per patient (*R*^2^, 0.99; wCOV, 11.2%).

**Conclusion:**

In this prospective test-retest setting, SUV parameters demonstrated high repeatability, in particular in LNs, while volumetric parameters demonstrated low repeatability. Further, the large number of lesions and wide distribution of SUVs included in this analysis allowed for the demonstration of a dependence of repeatability on SUV, with higher SUVs having more robust repeatability.

## 1. Introduction

Positron emission tomography (PET) with ligands targeting the prostate-specific membrane antigen (PSMA) is being increasingly utilized, with applications including treatment planning in patients with metastatic prostate cancer (PC) [1, 2]. The accessibility of the PSMA active site to high-affinity ligands, combined with rapid internalization, allows for accurate, noninvasive high-contrast imaging [3]. Given its facile synthesis without need for a cyclotron, ^68^Ga-labeled radiotracers have been, to date, widely used. However, recent years have also witnessed an increased use of ^18^F-labeled radiotracers, initially with ^18^F-DCFBC [4] and other first-generation compounds, and later more widely available radiotracers such as ^18^F-PSMA-1007, ^18^F-rhPSMA-7 [5, 6], and ^18^F-DCFPyL (piflufolastat F18, PYLARIFY®) [7]. The latter agent has been extensively investigated in major clinical trials [8, 9], including the multicenter phase 3 CONDOR and in the phase 2/3 OSPREY trials [10, 11], demonstrating positive predictive values of 78-91% in both detecting PC in pelvic lymph nodes (LN) and distant metastases. Based on the encouraging results, ^18^F-DCFPyL recently received approval from U.S. Food and Drug Administration (FDA) [12]. As a nationwide, commercially available, ^18^F-labeled PSMA PET agent [12], one may anticipate an increased use of this radiotracer in both clinical routine and for trials.

The repeatability of uptake features is an important property of ^18^F-DCFPyL to understand response assessment, e.g., in a theranostic setting or in men starting abiraterone or enzalutamide [8, 13]. If rigorously executed, standardization of imaging protocols and continuously calibrated PET devices allow for high test-retest repeatability [14], but biological aspects or interpatient and intrapatient variability can have a significant impact on quantitative features in repeated imaging studies [15].

In this regard, a recent study has reported high repeatability for 36 lesions in 12 patients using ^18^F-DCFPyL [16]. In this prospective clinical trial, we aimed to elucidate the repeatability of quantitative parameters on ^18^F-DCFPyL PET in a test-retest cohort by enrolling 21 men with PC with a total of 230 visible lesions. This relatively large cohort with a corresponding large number of disease sites enabled evaluation of repeatability among different organ compartments, such as in LN or osseous lesions, and among a wide range of SUVs. In addition, such an approach also allowed us to assess the dependence of SUV on original and relative units (in %) and to determine whether higher SUVs have improved repeatibility when compared to lower SUVs. This may be of importance for response assessment studies, where percentage change in SUV by comparing baseline and follow-up scans is a method to define progressive disease [17] or follow response in patients receiving PSMA-targeted radioligand therapy (RLT). Of note, higher SUVs from PSMA PET are linked to better early biochemical response [18] and overall survival [19] in patients under PSMA-directed treatment. Thus, the reader deciding on the appropriateness of RLT must have certainty on the reliability of these semiquantitative parameters.

## 2. Materials and Methods

This study was registered at ClinicalTrials.gov (NCT03793543) and was carried out under a United States FDA Investigational New Drug Application (IND121064). The Institutional Review Board of the Johns Hopkins Hospital approved this prospective study (IRB00174393).

### 2.1. Patients

Patient characteristics are displayed in Table 1. 21 patients with mean age 65.4 ± 9.4 years with history of PC were included in this trial. Among others, required inclusion criteria for the study were as follows: (1) age ≥ 18 years, (2) history of histologically or cytologically confirmed adenocarcinoma of the prostate without neuroendocrine differentiation, (3) patients with metastatic castration-sensitive or castration-resistant prostate cancer (CRPC) with evidence of metastatic disease on conventional imaging with computed tomography (CT) and/or bone scan, and (4) Eastern Cooperative Oncology Group performance status of ≤2 [14].

The exclusion criteria were as follows: (1) serious or uncontrolled coexistent nonmalignant disease, including active and uncontrolled infection; (2) administration of a radioisotope ≤ 5 physical half-lives prior to the first PET/CT; and (3) administration of an intravenous X-ray contrast medium ≤24 hours or oral contrast medium ≤120 hours prior to the first PET/CT.

### 2.2. Imaging Protocol

^18^F-DCFPyL was synthesized as previously described [7]. The imaging protocol followed current guidelines [20]. Patients were scanned in the supine position starting from the mid-thigh to the vertex of skull (whole body protocol) at approximately 60 min postinjection. PET/CT was obtained using a 128-slice Biograph mCT (Siemens Healthineers, Erlangen, Germany) with low-dose CT attenuation correction (no contrast, 120 kV, 40 effective mAs, 0.5 tube rotation time, and 0.8 pitch). Standard ordered-subset expectation maximization reconstructions with time-of-flight were used. A subsequent near-term ^18^F-DCFPyL PET/CT follow-up scan with identical imaging protocol was conducted to assess test-retest repeatability. No change in therapy occurred between the scans.

### 2.3. Image Analysis

A consensus central review was carried out with all images analyzed by three physicians with experience in the interpretation of PSMA-targeted PET/CT (BH, RAB, and RAW, having at least 3 years of experience in reading scans) who were blinded to clinical data. Images were analyzed using the InterView Fusion software (Version 3.08.005.0000, Mediso Medical Imaging Ltd., Budapest, Hungary) for lesion identification and segmentation.

As described in [21], the entire volume of all ^18^F-DCFPyL-avid tumor lesions (i.e., tumor burden) was manually segmented using volumes of interest. Mean and maximum standardized uptake values (SUV_mean_, SUV_max_) were assessed. In addition, tumor volume (TV) was computed, which allowed for calculation of total-lesion PSMA (TL-PSMA, defined as TV × SUV_mean_) [22].

### 2.4. Statistical Analysis

Corresponding uptake parameters were compared between both scans. Scatter diagrams were plotted, and linear regression analysis was performed. Bland-Altman plots were created for both absolute and relative differences of these data (expressed as a percentage), including upper and lower levels of agreement [23, 24]. For correlation of uptake, Pearson correlation was performed (providing *R*^2^). Kendall's tau (*τ*) was also used for correlational analyses with *τ* ≥ 0.40 indicating strong correlation [25, 26]. The within-subject coefficient of variation (wCOV, in %) was assessed [27]. For comparison of different wCOVs, the method of Forkmann was used [28]. A lesion-based head-to-head comparison including LN, osseous, and other lesions was conducted. Moreover, to assess for a dependence of the repeatability on different parameters, all lesions were subdivided into a group below (“< median”) vs. above (“> median”) the corresponding median value. In addition, the hottest lesion per patient (defined as metastatic site of disease with the highest SUV_max_ among all lesions) was also analyzed. A *P* value *<0.05* was considered statistically significant. Statistical analysis was performed with MedCalc software (Version 19.6, MedCalc software Ltd., Ostend, Belgium) and Microsoft Excel 2016 (Microsoft Cooperation, Redmond, WA, USA).

## 3. Results

### 3.1. Patients

**Figure 1:**
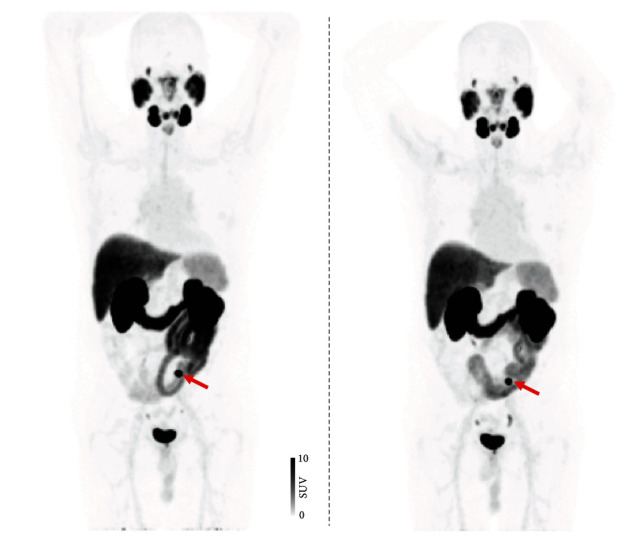
Test ^18^F-DCFPyL PET/CT (a) compared to retest ^18^F-DCFPyL PET/CT (b). A 59-year old patient afflicted with prostate cancer (Gleason Score 8) referring for staging (prostate-specific antigen level at time of scan, 1.0 ng/ml). Maximum intensity projections of both scans revealed identical DCFPyL-avid lymph node in the pelvis (red arrow).

**Figure 2:**
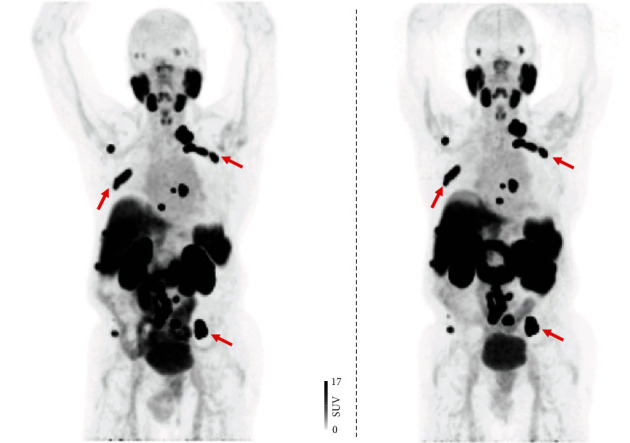
Test ^18^F-DCFPyL PET/CT (a) compared to retest ^18^F-DCFPyL PET/CT (b). A 88-year old patient afflicted with prostate cancer (Gleason Score 7) referring for staging (prostate-specific antigen level at time of scan, 69.55 ng/ml). Maximum intensity projections of both scans revealed an identical DCFPyL-avid lymph node in the skeleton, including the ribs and the pelvis (red arrows).

Between March 2019 and March 2020, 21 patients each underwent two scans with a median time between scans of 3.7 ± 3.0 days (range, 1 to 7 days). For the test scan, 322.2 ± 4.2 MBq (range, 310.8–326.7 MBq) were administered. For the retest scan, 323.5 ± 4.1 MBq (range, 310.1-328.6 MBq) were injected. A total of 230 PSMA-avid lesions were delineated, with 177/230 (77%) located in the skeleton, 38/230 (16.5%) in LN, and 15/230 (6.5%) in other soft tissue sites.  Figure 1 shows a test-retest scan of a patient with low and Figure 2 with high tumor burden. An overview of uptake parameters including SUV_max_, SUV_mean_, TL-PSMA, and PSMA-TV can be found in Table 2.

### 3.2. Analysis of Repeatability Parameters

**Figure 3:**
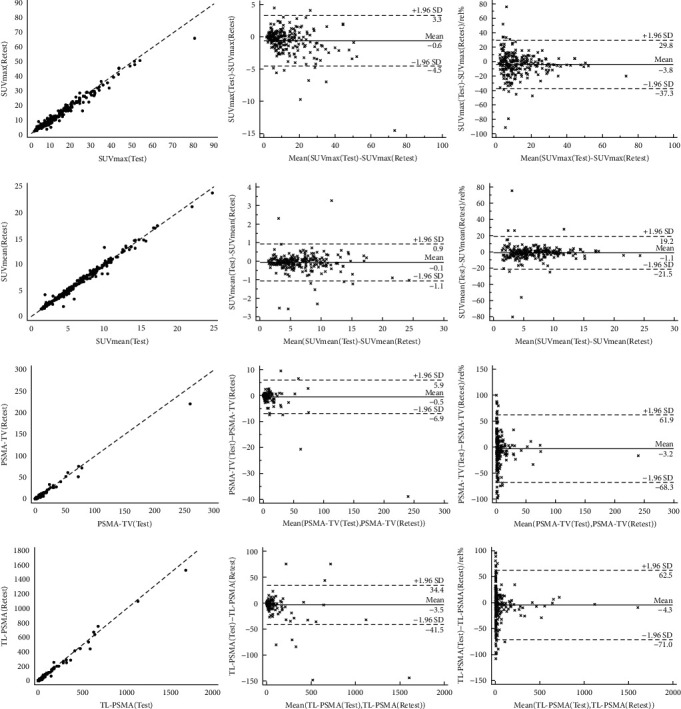
Correlation (first column), Bland-Altman for absolute values (second column) and Bland-Altman for relative values (third column) of quantitative parameters (first row, maximum standardized uptake values (SUV_max_); second row, mean standardized uptake values (SUV_mean_); third row, PSMA-avid tumor volume (PSMA-TV); and fourth row, total-lesion PSMA (TL-PSMA)). Good correlations were found for all parameters. Relative to SUV, volumetric parameters demonstrated larger magnitude of limits as presented by standard deviations on Bland-Altman plots for both absolute and relative values. The wide distribution of SUVs included in this analysis allowed for the demonstration of a dependence of repeatability on SUV, with higher SUVs having more robust repeatability, in particular for relative SUV_max_ values (top right).

For the entire tumor burden on the test scan, SUV_max_ was 13.1 ± 10.6 (range, 1.6–66.1) and SUV_mean_ was 6.7 ± 3.7 (range, 1.4–23.8), with almost identical results on the retest scan (SUV_max_, 13.7 ± 11.4 (range, 1.6–80.5); SUV_mean_, 6.8 ± 3.8 (range, 1.4–24.8)). The *R*^2^ values were ≥0.99 (Figure 3, first column; *τ*, SUV, ≥0.87; volumetric parameters, ≥0.83, *P* < 0.0001, respectively). Regardless which correlative analyses were applied, SUV_mean_ demonstrated the best correlation among all parameters (Table 2). wCOVs were high for SUV_mean_ (7.3%) and SUV_max_ (12.1%). For PSMA-TV and TL-PSMA, repeatability was lower (23.5% and 24.0%, respectively). Bland-Altman plots for all lesions are displayed in Figure 3, second and third columns. For both SUV_max_ and SUV_mean_, no systematic increase or decrease between the scans was noted (+/-1.96SD: 3.3/-4.5, 0.9/-1.1, respectively). Of note, higher SUVs had more robust repeatability, in particular for relative SUV_max_ values in % (Figure 3, top right). On Bland-Altman plots for PSMA-TV and TL-PSMA, larger magnitude of limits was recorded when compared to SUV (+/-1.96SD: 5.9/-6.9, 34.4/-41.5, respectively).

Lesions were subdivided into a group below vs. above the respective median value. Regardless of the investigated parameter, SUV derived from lesions above the median demonstrated a more robust repeatibility, in particular for SUV_mean_ (wCOV: SUV_mean_, >median, 4.1% vs. <median, 8.7%; SUV_max_, >median, 8.8% vs. <median, 16.6%; *P* < 0.0001, respectively; Supplementary Table).

Findings were similar with just the hottest lesion in each patient, with an *R*^2^ value of 0.99 for SUV (SUV_max_: wCOV, 11.2%; *τ*, 0.97; SUV_mean_: wCOV, 1.2%; *τ*, 0.97). No systematic increase or decrease was noted on Bland-Altman plots (SUV_max_, +/-1.96SD: 5.6/-8.5; SUV_mean_, +/-1.96SD: 0.47/-0.21; Supplementary Figure).

### 3.3. Repeatability Parameters on a Compartment-Based Level

When investigating different types of lesions, comparable *R*^2^ values were achieved for both LN (≥0.984) and lesions in the skeleton (≥0.988), which were slightly higher for SUV_mean_ (≥0.996). *τ* was ≥0.78. SUV_mean/max_ of LN and osseous lesions yielded high to intermediate repeatability, with significantly lower wCOV calculated for LN sites of disease (SUV_max_: LN 8.8% vs. skeleton 12.0%, *P* < 0.03; SUV_mean_: LN 3.8% vs. skeleton 7.8%, *P* < 0.0001). TV-based features again demonstrated low repeatability, with no significant differences between LN and osseous lesions (PSMA-TV: LN, 24.1% vs. bone, 22.8%, *P* = 0.63; TL-PSMA: LN, 23.5% vs. skeleton, 23.3%, *P* = 0.9; Table 2). Due to small number, visceral lesions were not analyzed further.

## 4. Discussion

230 lesions on 21 ^18^F-DCFPyL PET/CTs were utilized to demonstrate overall high repeatability of uptake. Volumetric features revealed relatively lower repeatability, while SUV_mean_ not only demonstrated the highest correlative indices (*τ*, 0.92-0.95) but also the best repeatability, in particular for LN (wCOV 3.8%). For SUV_max_, robust correlations along with at least intermediate repeatability were noted in LN and osseous lesions, suggesting SUV as a reliable metric for quantitative assessments. For ^18^F-DCFPyL PET, SUV-based parameters might be an acceptable alternative to volumetric parameters [8]. Importantly, we observed an improved repeatability for higher SUVs when considered relative to the level of uptake (relative units).

^18^F-DCFPyL is a U.S-wide, FDA-approved, PSMA-targeted, radiolabeled imaging agent for patients with PC [8, 9, 12] and a more worldwide use can be anticipated, indicating the importance of a thorough understanding of this agent. The high repeatability of uptake parameters, both overall and based on metastasis type, is of importance, as it suggests that ^18^F-DCFPyL may be useful for therapy response assessment and also that manual and automated (e.g., artificial intelligence) methods for lesion detection should be repeatable and reliable [13, 29, 30].

Previous studies have revealed comparable correlations and repeatability, but differences relative to the present trial must be noted. For instance, in a preceding analysis based on ^68^Ga-PSMA PET in a test-retest setting [31], the authors reported substantially higher wCOV, e.g., for SUV_mean_ derived from LN. Further, no significant differences between lesion type were observed with the ^68^Ga-labeled PSMA imaging agent [31]. This may be partially explained by the improved diagnostic accuracy of radiotracers labeled with ^18^F [32]. Intrinsic physical factors of ^68^Ga may contribute to the partial volume effect, which in turn has an impact on semiquantitative values such as SUV [33], potentially explaining such different wCOVs.

A recent study by Jansen et al. also reported on test-retest properties for ^18^F-DCFPyL, including a total of 36 lesions [16]. Similar to our findings, SUV_mean_ had a better repeatability when compared to SUV_max_ [16]. However, no significant differences between LN and osseous lesions were identified in the previous trial, but a trend towards significance was noted (*P* = 0.06) [16]. In our study, significant differences between lesions located in the skeleton and LN were determined, possibly due to the increased number of subjects and lesions [16]. In this regard, relative to the investigation of Jansen et al. [16], more lesions were included (230 vs. 36) providing a broad range of SUV (1.4–80.5). This allows us to demonstrate a dependence of repeatability on SUV, with higher SUVs having a higher repeatability, in particular for relative SUV_max_ values (Figure 3, third column). This observation is of importance, as absolute SUVs have different ranges depending on their normalization schemes, whereas relative differences allow for intra- and interindividual comparisons [34]. In addition, this marked dependence of SUV on relative units may be clinically relevant, e.g., for response assessment studies, where it is common to indicate percentage change in SUV by comparing baseline and follow-up scans, as recently demonstrated for ^18^F-DCFPyL [17]. Assessment of delta % has also been recently suggested by the PSMA PET Progression Criteria, with an increase in PSMA uptake of 30% indicating progressive disease [35]. As such, the observed improvement of relative repeatability at the higher SUVs may be important for future multicenter trials, e.g., for ^18^F-DCFPyL-based therapy response monitoring [8] or for patients scheduled for RLT.

In this regard, no study to date has explored the predictive potential of ^18^F-labeled PSMA PET for subsequent outcomes in patients with PC scheduled for PSMA-directed therapy [36]. The repeatability of SUV_max_ units demonstrated in this study may lay the foundation for future investigations of the utility of ^18^F-DCFPyL PET in monitoring ^177^Lu-based RLT. These considerations are further fueled by the fact that in patients scheduled for PSMA-targeted RLT, high average SUVs on baseline PSMA PET are frequently observed (up to 73.4) and that increased baseline SUV_max_ were linked to improved early biochemical response (cut-off, >19.8) [18] and overall survival (cut-off, >14.3) [19]. In this analysis, the highest SUV_max_ was 80.5, and thus, the results are relevant to the patient population undergoing RLT. The higher repeatability at higher SUV_max_ may be of importance in the theranostic setting, as the reader deciding on RLT has certainty that such findings are not related to measurement variability, suggesting SUV_max_ is a reliable imaging biomarker to identify high risks prone to treatment failure. This also applies regardless if lesions are located in the skeleton (Figure 2) or LN (Figure 1), as repeatability of SUV_max_ was high to intermediate among metastases allocated to different organ compartments (Table 2).

Both ^68^Ga- and ^18^F-labeled, PSMA-directed radiotracers demonstrate that the best repeatability is found with SUV, whereas values for TV may have to be interpreted with caution [16, 31]. As a possible explanation, the latter parameter may be subject to an operator-dependent bias of manual segmentation. Fully automated delineation software may increase repeatability, e.g., when artificial intelligence such as deep learning is applied [37]. Moreover, state-of-the-art reconstruction algorithms such as point-spread function (PSF) may also recategorize lesions as more definitive sites of disease attributable to PC, as recently demonstrated for ^18^F-DCFPyL [38]. However, the effect of PSF on repeatability in patients scheduled for ^18^F-DCFPyL has also been reported, with PSF reconstruction significantly having a negative impact on repeatability for SUV, but not for TV [16]. Given these contradictory results of increased interpretative certainty and decreased repeatability by implementing PSF, future studies should explore the impact of novel and advanced reconstruction algorithms on test-retest metrics.

This study has several limitations. Although providing the largest cohort of patients and lesions to date, some patients had a disproportionate number of lesions and clustering effects from that lesion distribution may have effected the results. Therefore, a hottest lesion analysis investigating the metastatic site with the highest SUV_max_ per subject was also performed. Again, a high repeatability with no systematic increase or decrease was noted (Supplementary Figure), further corroborating the findings including all suspected sites of disease. Moreover, lesion size, dose, and patient factors including interpatient and intrapatient variability can have a significant impact on semiquantitative assessments using this radiotracer [15, 39]. Therefore, future studies should also consider controlling for such day-to-day variables [16]. Partial volume effects are almost certainly a factor in repeatability in small lesions, and future test-retest studies might exclusively enroll patients with extensive tumor burden. Such an approach would then corroborate our present findings across a broad spectrum of tumor burden. Despite enrolling the largest cohort of patients in a prospective test-retest setting for ^18^F-DCPFyL to date, the number of patients with different therapies was too small to provide reliable results for a subanalysis focusing on prior therapeutic regimens. This should also be addressed in future studies.

## 5. Conclusion

Our results demonstrate that ^18^F-DCFPyL has highly repeatable uptake parameters in PC lesions. Further, the large number of lesions and wide distribution of SUVs included in this analysis allowed for the demonstration of a dependence of repeatability on original and relative SUVs, with higher SUVs having more robust repeatability. This observed improvement of repeatability at increased SUVs may be important for future multicenter trials, e.g., for ^18^F-DCFPyL-based response monitoring in patients under antihormonal treatment.

## Figures and Tables

**Table 1:** Patient's characteristics.

Age (mean ± SD, in years)	65.4 ± 9.4
Height (mean ± SD, in m)	1.78 ± 0.08
Weight (mean ± SD, in kg)	92.4 ± 18.1
PSA level in ng/ml, mean ± SD (range)	22.3 ± 34.3 (0.4-138.4)
Prior therapies (numbers in parentheses indicate %)	
In total	19/21 (90.5)
Surgery	13/21 (61.9)
Hormonal therapy	19/21 (90.5)
RTx	14/21 (66.7)
CTx	9/21 (42.9)

SD: standard deviation; CTx: chemotherapy; PSA: prostate-specific antigen; RTx: radiation therapy.

**Table 2:** Head-to-head comparison of semiquantitative parameters for both scans, for all lesions (*n* = 230), osseous (*n* = 177), and lymph node lesions (*n* = 38), mean value and standard deviation along with respective Pearson correlation, Kendall's tau (*τ*), and within-subject coefficient of variation (wCOV).

	Test	Retest	*R* ^2^	Kendall's *τ*	wCOV (%)
All lesions (*n* = 230)					
SUV_max_	13.1 ± 10.6	13.7 ± 11.4	0.988	0.87	12.1
SUV_mean_	6.7 ± 3.7	6.8 ± 3.8	*0.996*	*0.93*	*7.3*
PSMA-TV	6.3 ± 17.7	6.8 ± 20.2	0.987	0.83	23.5
TL-PSMA	58.7 ± 161.7	62.2 ± 170.5	0.991	0.85	24.0
Osseous lesions (*n* = 177)					
SUV_max_	13.4 ± 11.3	14.3 ± 12.1	0.990	0.87	12.0
SUV_mean_	6.6 ± 3.8	6.7 ± 3.8	*0.996*	*0.92*	*7.8*
PSMA-TV	7.3 ± 20.1	7.9 ± 22.8	0.988	0.85	22.8
TL-PSMA	68.1 ± 181.6	62.5 ± 191.0	0.991	0.86	23.3
Lymph node lesions (*n* = 38)					
SUV_max_	14.2 ± 7.6	14.1 ± 8.2	0.984	0.86	8.8
SUV_mean_	8.3 ± 3.3	8.2 ± 3.2	*0.996*	*0.95*	*3.8*
PSMA-TV	3.3 ± 4.4	3.3 ± 5.0	0.987	0.78	24.1
TL-PSMA	34.4 ± 56.9	35.8 ± 64.5	0.994	0.86	23.5

Regardless which statistical test was used, mean standardized uptake value (SUV_mean_) achieved the highest correlative indices (Pearson correlation, Kendall's *τ*) and the lowest wCOV, indicating excellent repeatability, in particular for lymph node disease (marked in italic). Volumetric features, however, revealed lower *τ* and still acceptable repeatability, as indicated by increased wCOV. SUV_max_: maximum standardized uptake value; PSMA-TV: PSMA tumor volume; TL-PSMA: total lesion PSMA.

## Data Availability

The data are not publicly available because, due to the European regulations regarding data protection, we cannot make data available online or disburse them. However, all data are available for revision on-site.

## Supplementary Materials

Supplementary Materials Supplementary Figure: hottest lesion analysis in a test-retest setting, with correlation of (A) maximum standardized uptake values (SUV_max_), (B) mean standardized uptake values (SUV_mean_), and (C, D) corresponding Bland-Altman plots. An excellent correlation between test and retest scans along with a considerable low magnitude of limits within standard deviations (SD) was noted. Supplementary Table: differences in within-subject coefficient of variations (wCOVs, in %) for all parameters, divided into a group below (<) vs. a group above (>) the corresponding median value (*n* = 115 per group). Regardless of the investigated parameters, lesions above the median had a more robust repeatibility, which was markedly better for standardized uptake value (SUV), in particular for SUV_mean_. SUV_max_: maximum SUV; PSMA-TV: PSMA tumor volume; and TL-PSMA: total lesion PSMA. *P* has been derived from comparison of wCOV from lesions below vs. above the respective median. SD: standard deviation.
