# Supplementary material for: High SUVs Have More Robust Repeatability in Patients with Metastatic Prostate Cancer: Results from a Prospective Test-Retest Cohort Imaged with 18F-DCFPyL
Source: Mol Imaging. 2022 Feb 23;2022:7056983. doi: 10.1155/2022/7056983 (PMC8896803; doi:10.1155/2022/7056983)
Supplement: Supplementary Materials — Supplementary Figure: hottest lesion analysis in a test-retest setting, with correlation of (A) maximum standardized uptake values (SUVmax), (B) mean standardized uptake values (SUVmean), and (C, D) corresponding Bland-Altman plots. An excellent correlation between test and retest scans along with a considerable low magnitude of limits within standard deviations (SD) was noted. Supplementary Table: differences in within-subject coefficient of variations (wCOVs, in %) for all parameters, divided into a group below (<) vs. a group above (>) the corresponding median value (n = 115 per group). Regardless of the investigated parameters, lesions above the median had a more robust repeatibility, which was markedly better for standardized uptake value (SUV), in particular for SUVmean. SUVmax: maximum SUV; PSMA-TV: PSMA tumor volume; and TL-PSMA: total lesion PSMA. P has been derived from comparison of wCOV from lesions below vs. above the respective median. SD: standard deviation. [file 7056983.f1.zip › Supplementary Table.docx]

|  | wCOV  > median | wCOV  < median | *P* |
| --- | --- | --- | --- |
| *SUV_max_* | 8.8 | 16.6 | <0.0001 |
| *SUV_mean_* | 4.1 | 8.7 | <0.0001 |
| *PSMA-TV* | 16.4 | 21.5 | 0.006 |
| *TL-PSMA* | 16.7 | 39.1 | <0.0001 |

**Supplementary Table. Differences in within-subject coefficient of variations (wCOVs, in %) for all parameters, divided into a group below (<) vs. a group above (>) the corresponding median value (n=115 per group).** Regardless of the investigated parameters, lesions above the median had a more robust repeatibility, which was markedly better for standardized uptake value (SUV), in particular for SUV_mean_. SUV_max_ = maximum SUV. PSMA-TV = PSMA tumor volume and, TL-PSMA = total lesion PSMA. *P* has been derived from comparison of wCOV from lesions below vs. above the respective median. SD=standard deviation.
